# A Method for Simultaneous Polishing and Hydrophobization of Polycarbonate for Microfluidic Applications

**DOI:** 10.3390/polym12112490

**Published:** 2020-10-27

**Authors:** Dominika Ogończyk, Paweł Jankowski, Piotr Garstecki

**Affiliations:** Institute of Physical Chemistry, Polish Academy of Sciences, Kasprzaka 44/52, 01-224 Warsaw, Poland; pjankowski@ichf.edu.pl

**Keywords:** microfluidics, polycarbonate, surface modification, polishing, hydrophobization

## Abstract

Here we present a new methodology for chemical polishing of microchannels in polycarbonate (PC). Tuning the time of exposition and the concentration of ammonia, the roughness arising from the micromachining process can be significantly reduced or completely removed while preserving the structure of microchannels. Besides smoothing out the surface, our method modifies the wettability of the surface, rendering it hydrophobic. The method increases the optical transparency of microchannels and eliminates undesired effects in two-phase microfluidic systems, including wetting by aqueous solutions and cross-contamination between aqueous droplets that could otherwise shed satellites via pinning.

## 1. Introduction

Progress in microfluidics has been directly reliant on the access to facile methods for microfabrication. Micromilling is one of the most commonly used methods for fast prototyping of polymeric microfluidic devices. In the process, parts of originally flat, and typically smooth material are removed mechanically with a spinning milling bit. As the milling bit moves over the machined surface while spinning, it leaves finite roughness of the surface. The magnitude of the roughness depends on the process parameters such as: spindle speed, cut depth, feed rate and working environments [1]. Typically, the roughness of PC microchannels depends on the size of the used cutter and can be tamed under 1 µm for cutters of diameter between 0.1 and 3 mm [2]. Such roughness is typically undesired, because it decreases the optical transparency of the walls of the microchannels, may cause increased slip at the walls, as compared to the smooth microchannels [3]. In droplet applications, the most important effect is in the rough edges causing pinning of the liquid-liquid interface of a passing droplet, making it more difficult to control the flow and to avoid the wetting of the walls by the droplet liquid, and potentially leading to cross-contamination between droplets [4].

The surfaces of thermoplastics may be polished. The commonly used methods include: (i) mechanical polishing, (ii) fire polishing, (iii) laser melting and (iv) chemical vapor polishing [4]. Due to the tightly restricted physical access to the surfaces of microchannels mechanical polishing is difficult to apply. Fire polishing renders itself difficult to be applied controllably in the microchannels. The most suitable approach to polishing the surfaces of microchannels is via chemical methods. Some methods for smoothing out the surface of microchannels made in polycarbonate have already been proposed, including the use of thermal-flow technique [5] and CH_2_Cl_2_ vapor polishing [4]. The heat treatment (10–20 °C below the glass transition temperature) reduces the viscosity of the polymer and enhances its fluidity and yields smoothing of the surface corrugation of the waveguide without significantly altering the shape of the microstructures. In turn, vapor PC polishing is based on directing hot stream of vaporized CH_2_Cl_2_ onto the surface of the selected part of the chip (modification of elements before bonding process of the chip). It is worth to note that these two methods are easily used for polishing of outer surface of PC elements [4,5], however, the use of these methods inside microfluidic devices can be limited due to difficult access of the active agent (heat, vapours) to the interior part of the channel. In contrast to all of the methods referenced above, the simple protocol that we propose here enables direct polishing of milled surfaces inside polycarbonate microchannels.

The second important aspect of the character of the surface of PC is its wettability. The wettability of native PC surface (contact angle is 84°) into a hydrophobic or even superhydrophobic material is possible via physical [6,7,8,9,10,11,12,13,14,15,16] or chemical modification [17]. The physical approach uses surface texturing via controlled laser ablation [9,10,14] or via solvent induced crystallization of polycarbonate [6,7,8,11,12,13,15,16]. To put it another way, a hierarchical surface structure obtained by physical modification (texturing geometry and depth) strongly increases the surface roughness [18,19]. Different types of hierarchical structures (pores, cavities and pillars) can be fabricated with the use of lasers, which consequently yields increasing the PC static contact angle from 84° to 120° [9,14] or even 155° [10]. Another physical approach employs a phase separation process with the use of chemical solvents such as: dichloromethane [8], acetone [7,11,12,16], dimethylformamide [6] or a mixture of the last two [13,15]. The resulting surface texture depends on the parameters of the process such as: the type and concentration of solvent, temperature and process duration. In general, crystallization leads to the formation of microporous spherulites covered with nanofibrils structures and produces superhydrophobic wetting behavior. Physical modifications of PC transformation yield hydrophobic surfaces with high chemical stability and good self-cleaning properties. Nevertheless, optical transparency of PC after the surface crystallizations is lowered via scattering on the surface texture. Finally, PC can be rendered hydrophobic using a chemical reaction with the use of an amine with a long alkyl chain that easily reacts with PC carbonate groups to create urethane bond [17,20]. This process not only drapes the surface with hydrophobic alkyl chains of the amine but also modifies the topography of the surface increasing its roughness.

In this paper we describe a simple method that enables polishing of the surfaces of polycarbonate microchannels altered after assembly of the device. The method homogenizes the topography and surface properties of all the walls of the channels, both milled and native. In addition, the method increases the hydrophobic character of the surface, further decreasing the propensity of aqueous droplets to wet the channel walls. The procedure is simple as it only requires flushing the channels with an aqueous ammonia solution at ambient temperature.

For additional clarity, we present the comparison of the known in the literature methods of polycarbonate polishing and hydrophobization with the proposed methodology (Table 1).

## 2. Materials and Methods

We used ammonia aqueous solution 29% pure p.a. (Chempur, Piekary Śląskie, Poland), Fluorinerts FC-40 and Novec fluid HFE-7500 (3M), squalane, mineral oil (light), poly(dimethylsiloxane) 50 cSt and 10 cSt (Sigma-Aldrich, Darmstadt, Germany). All chemicals were pure and analytical grade and were used without further purification. Bacterial nutrients: Mueller Hinton Broth 2 (MH2; Sigma Aldrich) and Brain Heart Infusion LAB-AGAR™ (BHI; Biocorp, Issoire, France) were used. For the preparation of the aqueous solutions we used Milli-Q water.

**Figure 1:**
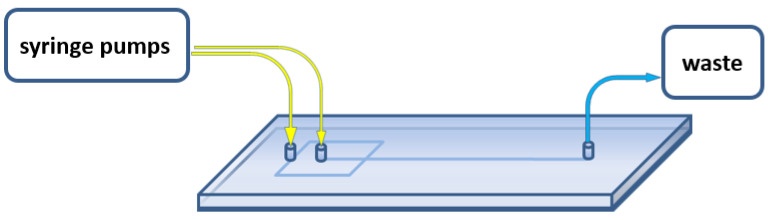
The experimental setup comprises the needed amount of syringe pumps, the polycarbonate chip, and a waste container. In the optimal conditions, the microchannels are modified with the use of 20% NH_4_OH for 2 h with flow rate equals 6 mL h^−1^ and then are rinsed with water for 30 min with the same flow rate.

In experiments and measurements e.g., contact angles we used polycarbonate plates (Makrolon, Bayer) of sizes 40 × 20 × 0.75 mm. We fabricated the microfluidic chips in polycarbonate by micromilling (Ergwind MSG4025, Sopot, Poland) in plates of thickness of 5 mm and then bonded them with plane 2 mm plates. In the procedures of PC chips modification we used syringe pumps (PHD 2000, Harvard Apparatus, Holliston, MA, USA) (Figure 1). The microchannels were modified with the use of 20% NH_4_OH for 2 h with flow rate equals 6 mL h^−1^ and then were rinsed with water for 30 min with the same flow rate. The flow rate of ammonia solution has a negligible impact on the quality of smoothing and hydrophobization. Still, we noted that: (i) too slow flow may result in the undesirable emission of ammonia gas bubbles, and (ii) too fast flow causes excessive consumption of reagent. For this reason, we considered the flow of 6 mL h^−1^ to be optimal.

### 2.1. Contact Angle Measurements

Contact angle (CA) measurements were done with the use of the static sessile drop method. In the case of CA of water in air (water/PC/air) and in oil (water/PC/oil) we took images of a 2 µL droplet of distilled water on the surface. We used a camera (UI-1120SE, uEye, Obersulm, Germany) to record the shape of the droplet and measured the value of the CA with ImageJ (Fiji) application (National Institute of Mental Health, Bethesda, MD, USA).

### 2.2. Surface Analysis

High magnification SEM images were captured with the use of the field emission Nova NanoSEM™ 450 scanning electron microscope (Thermo Fisher Scientific, Waltham, MA, USA).

## 3. Results and Discussion

**Scheme 1:**
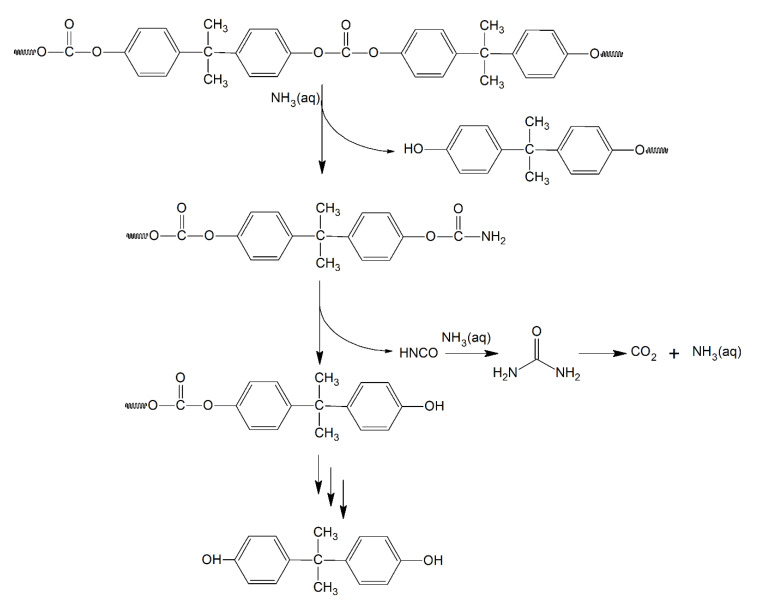
Based on [21], we present a scheme of the presumable mechanism of the PC monomerization with the use of NH_3(aq)_.

Ammonia enables monomerization of PC. The mechanism behind this process is similar to an hydrothermal (at temperature ranging from 433 to 463 K and at a pressure of 10 MPa) chemical recycling of polycarbonate [21] and involves monomerization of PC in the following manner (Scheme 1): carbonate bonds are degraded and carbamate esters are formed, that are further decomposed into isocyanates and polymers with terminal group of bisphenol A. Next, the isocyanate reacts with ammonia to produce urea, that is further decomposed into carbon dioxide and ammonia. In turn, by repeating the above mechanism the polymers are finally degraded to bisphenol A. Interestingly, ammonia reacts with PC not as an alkaline reagent (i.e., not as the ammonia ions) but as a nucleophile.

**Figure 2:**
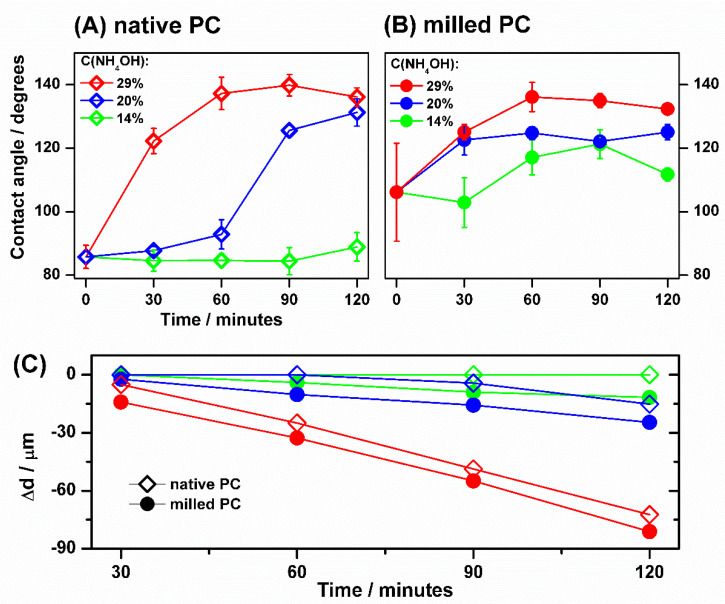
Contact angle of water on polycarbonate modified in ammonia solution with various concentrations (14—green, 20—blue and 29%—red) in the function of time of exposure for native PC (**A**, diamonds) and completely milled PC slabs (**B**, circles). The values of the contact angle are the averages of three measurements. (**C**) Effect of the ammonia solution concentration and duration of modification of native PC (diamonds) and completely milled PC slabs (circles) in the function of thickness of removed layer of PC surface.

We hypostatized that the chemical treatment under mild, yet not hydrothermal conditions can be useful for polishing of micromachined structures of polycarbonate microchannels with synchronous hydrophobization of the surface. In contrary to the complete conversion to bisphenol A in hydrothermal conditions, in our experiments we verified if PC is also slightly monomerized (evidenced by the removal of a surface layer of polycarbonate) by aqueous ammonia solution. In order to optimize the method we tested the influence of the concentration of ammonia and of the duration of the reaction. We performed experiments with the use of native and milled slabs of PC (Figure 2). The tested range of concentration of water solution of ammonia was from 14% to 29%. We also varied the time of exposition between 30 min and 2 h.

**Figure 3:**
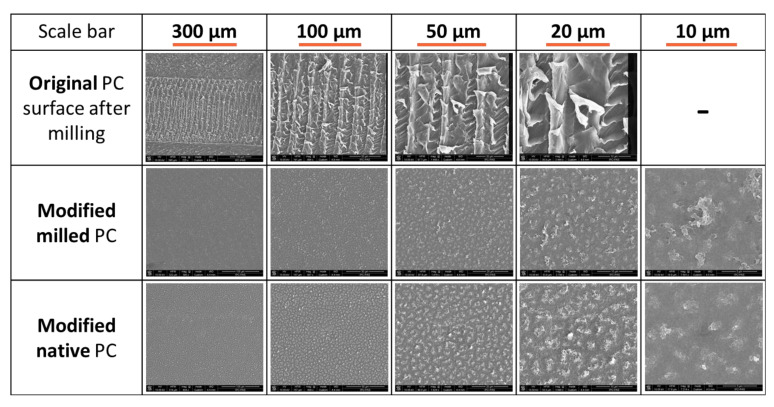
Scanning electron micrographs of original and modified PC with the use of 20% solution of NH_4_OH for 2 h at room temperature.

We found that contact angle (CA) of modified PC depends strongly on both parameters: ammonia concentration and duration of the reaction. In general, the hydrophobicity increased with concentration of the reagent and reaction time. We observed that the rate of the modification depends not only on time and concentration of the reagent but also on the type of the PC surface: native (Figure 2A) or milled (Figure 2B). In the consequence of the test of these two types of surfaces we claim that the milled PC is removed easier and in comparison with native PC the thickness of the removed layers is close to 10 micrometers higher in identical conditions of the modification process. It seems that the difference that we observed is a consequence of the presence of a protective layer on the surface of the native PC and the difference in initial surfaces roughness (Figures S2 and S3). Nevertheless, the effect of surface roughness of modification with the use of 20 or 29% NH_4_OH for 2 h is almost the same for both types of PC surfaces (Figure 2 and Figure 3). However, we note here, that using of the 20% concentration of ammonia solution yields four times smaller change of a thickness of the removed layer in comparison with 29% NH_3(aq)_. Thus, in order to transform: native PC (contact angle is 85.8° +/− 3.7°) and milled PC (106.1° +/− 15.5°) into a more hydrophobic material (134.2° +/− 2.0°), it is necessary to use aqueous solution of ammonia in the concentration of 20–30% that digests the initial surface roughness and introduces the appropriate hierarchical surface structure (Figure 3, Figures S2 and S3).

**Figure 4:**
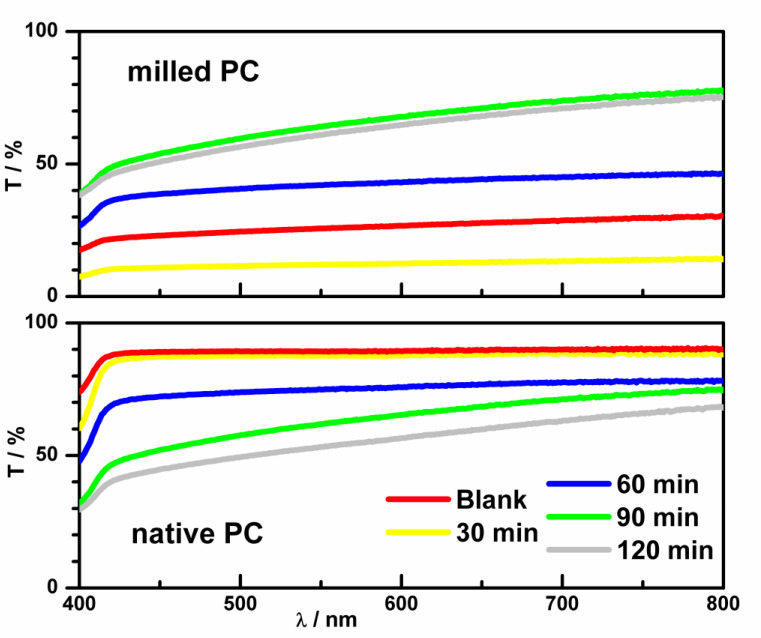
UV-VIS transmission spectra of the milled and native PC plates modified via exposure to 20% solution of NH_4_OH for 2 h at room temperature.

**Figure 5:**
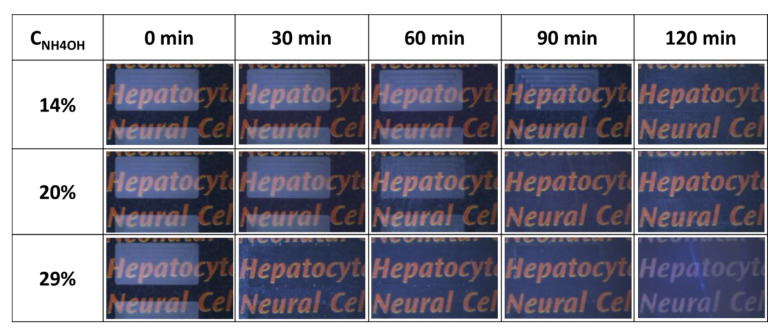
Comparison of optical transparency for native and micromachined (milled rectangles) surfaces of polycarbonate before and after modification with solutions of ammonia of a range of concentrations, over a range of intervals.

We assume that the optimum treatment should smoothen the surface yet leave the cross-section of the channel unmodified. For this reason, in further analysis our attention has been focused on PC modification with the use of 20% ammonia solution for 2 h, as it digests only about 15 or 25 micrometers of the native and milled PC surface, respectively. In Figure 4 we presented the results of spectral analysis for PC slabs that were modified with 20% NH_4_OH in different time. As shown in Figure 4 and Figure 5, the chemical modification caused the significant increase of optical transparency for the milled PC (with machined structures) and a slight decrease for native PC. It is worth highlighting that the initial transparency difference for the PC native and milled surfaces disappeared after the proposed PC modification. Nevertheless, the transparency of all types of PC surfaces after modification with the use of 20% NH_4_OH for 2 h is similar, just as we observed that the CAs did not differ between the originally native or milled surfaces after treatment (Figure 2). Additionally, we showed data sets of the spectral analysis of PC plates for other concentrations of NH_4_OH (14 and 29% in Figure S1 and Figure 5).

In general, the PC transparency can be altered with the use of NH_3(aq)_ treatment and increased with concentration of the reagent and/or reaction time (Figure 5). There is a very tight correlation between these two parameters and the optical transparency of the modified PC and using a higher NH_3(aq)_ concentration (from 14 to 29%) shortens the time of modification (from 120 to 30 min).

**Figure 6:**
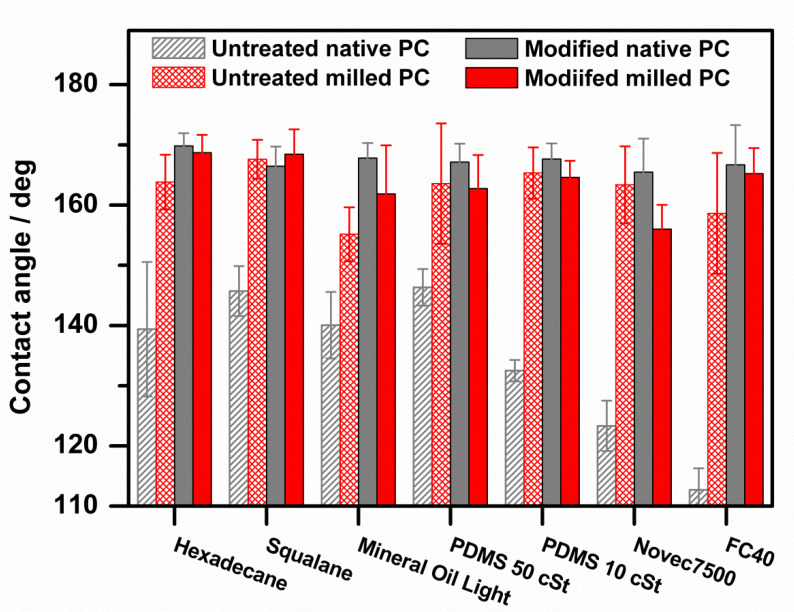
Contact angle (CA) of water drops deposited on the untreated and NH_3(aq)_ modified PC slabs (details in Figure) in surrounding various oils. CA was measured through the water degrees at the oil/water/solid interface. The volume of water droplets was 2 µL.

Furthermore, in order to verify the applicability of the modified PC surfaces to formation of droplets in microfluidic devices, we measured the contact angle of water drops deposited on the modified slabs in the continuous phase of various oils (Figure 6).

The resulting hierarchically structured surfaces of modified PCs (Figure 3, Figures S2 and S3) displayed CAs of water in oils above 160°, thus the modification affects the wetting properties (Figure 6). It is worth noting that changes in the contact angle values are much more pronounced for modified PC native surfaces than for modified PC milled surfaces. In turn, the changes in CAs of water in the air were more spectacular (Figure 2). Nevertheless, the use of the proposed modification of PC surfaces enables the production of the microfluidic systems to stable generate and transport aqueous droplets in microchannels. Additionally, we tested an oil wettability of the modified PC slabs and can conclude that they are superlipophilic (CA < 5°).

**Figure 7:**
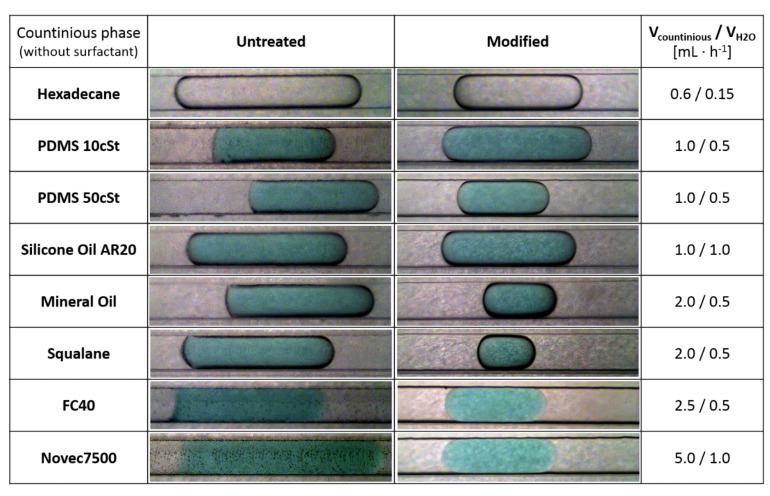
Result of the modification of PC in microchannels to elimination of wetting effect. Three milled walls and one native wall of microchannels were modified with the use of 20% NH_4_OH for 2 h with flow rate equals 6 mL h^−1^.

Further, we verified the utility of microchannels modified under optimal conditions for production of water droplets in various oils continuous phase (Figure 7). We tested the formation of water droplets in various oils (hexadecane, Squalane, PDMS, Silicone, and Mineral Oils) or fluorocarbon oils (FC-40 and Novec7500).

For all types of oils, we noticed elimination of wetting effect (Figure 7) and did not observe any evidence of wetting of the modified surface by the oil phase in the experiments conducted over a period longer than 6 h each.

**Figure 8:**
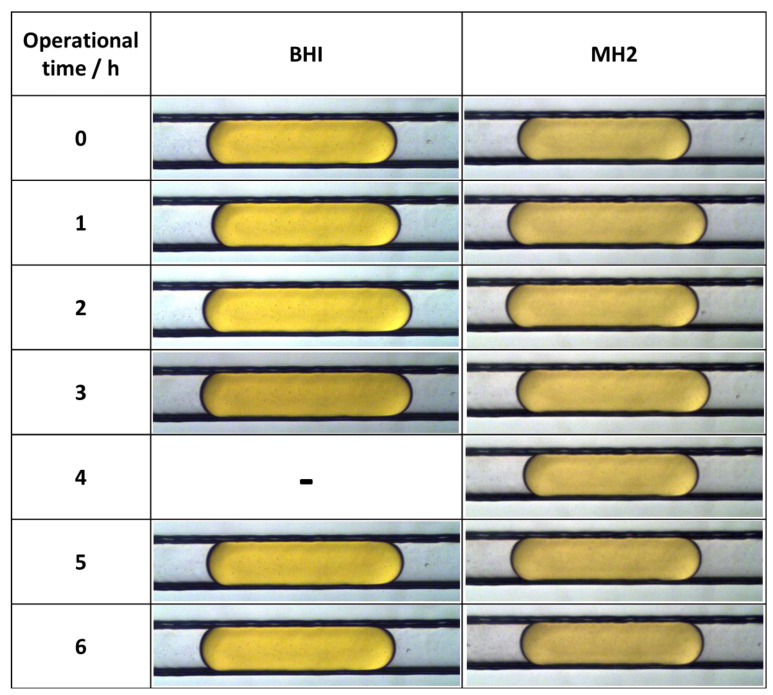
The stability of NH_3(aq)_ modified surface of PC microdevice was tested for 6 h of continuous work. During the test we used Novec 7500 with 1% PFPE-PEG-PFPE as a continuous phase and BHI and MH2 as an aqueous phase in flow-focusing microsystem.

Finally, we confirmed the stability of the generation of droplets of media for bacterial growth (BHI and MH2) in a flow-focusing microfluidic device modified with NH_3(aq)_ (Figure 8). The liquid bacterial media, which contain nutrients and physical growth components, have very complex chemical compositions and can significantly affect wetting effects. We continuously produced droplets of BHI and then MH2 for the minimum total time of 6 h. We did not observe any changes over time in the quality of the produced droplets.

## 4. Conclusions

In this paper we presented a new methodology of chemical polishing of microchannels that guarantees an optimal balance between the smooth surface finish and preservation of the machined microstructure. The process of PC modification is very simple and easy controllable by parameters such as: concentration of ammonia solution and time. We established that the optimum treatment which should smoothen the surface is the use of 20% ammonia solution for 2 h. Simultaneously, our method enables production of not only smooth but also hydrophobic surface of PC e.g., of channels in microfluidic devices. The results of polishing of micromachined structures of polycarbonate slabs and microchannels include: (i) the increase of optical transparency, (ii) the change of contact angle or/and (iii) wetting properties of the surface of microchannels.

## Figures and Tables

**Table 1:** Comparison of the polishing and hydrophobization methods of polycarbonate surface.

Method	Ref	Modification	Polishing	Hydrophobization	Useful in Microchannels
**Vapor Polishing**	[4]	with the use of dichloromethane	yes	no	no
**Thermal-Flow Technique**	[5]	via annealing at 10–20 °C below the glass transition temperature	yes	no	no
**Laser Surface Texturing**	[9]	via a controlled CO_2_ laser ablation	no	yes	no
	[14]	via a direct laser interference patterning	no	yes	no
**Thermal Surface Texturing**	[10]	via thermal replication of microtextured with the use of the Si master	no	yes	no
**Solvent Crystallization**	[6]	via an evaporation of *N*,*N*-dimethylformamide	no	yes	no
	[7]	via acetone-induced crystallization	no	yes	no
	[8]	via a CH_2_Cl_2_ dipping and coating by trichloromethyl silane sublimation	no	yes	no
	[11]	via a liquid acetone immersion	no	yes	no
	[13,15]	via acetone/DMF mixture treatment	no	yes	no
**Chemical Modification**	[17]	with the use of 5% ethanolic solution of dodecylamine	no	yes	yes
	[16]	with the use of: acetone, n-pentane and 1H,1H,2H,2H-perfluorooctyl trichlorosilane	no	yes	yes
**Solvent Surface Texturing**	**our work**	**with the use of NH_4_OH**	**yes**	**yes**	**yes**

## Supplementary Materials

The supplementary materials are available online at https://www.mdpi.com/2073-4360/12/11/2490/s1, Figure S1 UV-VIS transmission spectra of the milled (A,B) and native (C,D; untreated) PC plates modified via exposure to 14 (A,C) and 29% (B,D) solution of NH4OH for 2h at room temperature; Figure S2 Scanning electron microscopies of milled PC slabs that were modified with the use of various solutions of NH4OH for various times at room temperature; Figure S3 Scanning electron microscopies of native PC slabs that were modified with the use of various solutions of NH4OH for various times at room temperature.
