# Supplementary material for: A Method for Simultaneous Polishing and Hydrophobization of Polycarbonate for Microfluidic Applications
Source: Polymers (Basel). 2020 Oct 27;12(11):2490. doi: 10.3390/polym12112490 (PMC7692113; doi:10.3390/polym12112490)
Supplement: Supplementary file 1 [file polymers-12-02490-s001.pdf]

Supplemental information

Title: **Method for simultaneous polishing and hydrophobization of polycarbonate for microfluidic applications**

Authors: D. Ogończyk†,*, P. Jankowski† and P. Garstecki*

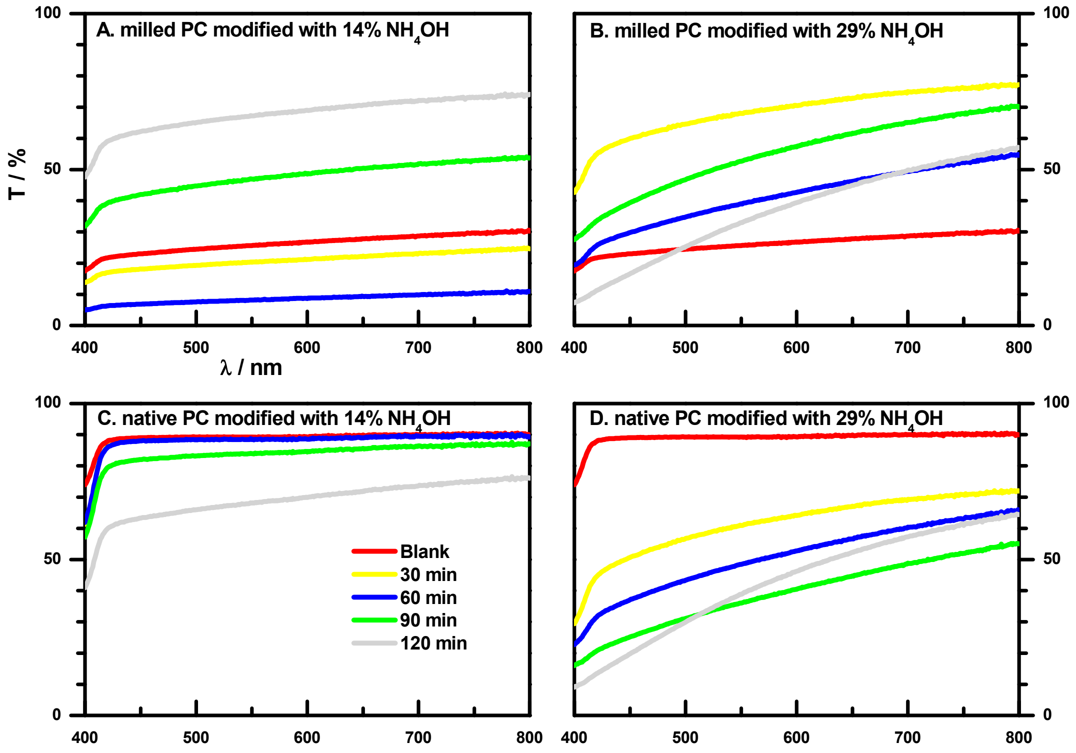

Figure S1 *UV-VIS transmission spectra of the milled (A,B) and native (C, D; untreated) PC plates modified via exposure to 14 (A, C) and 29% ((B, D) solution of NH_4OH for 2h at room temperature.*

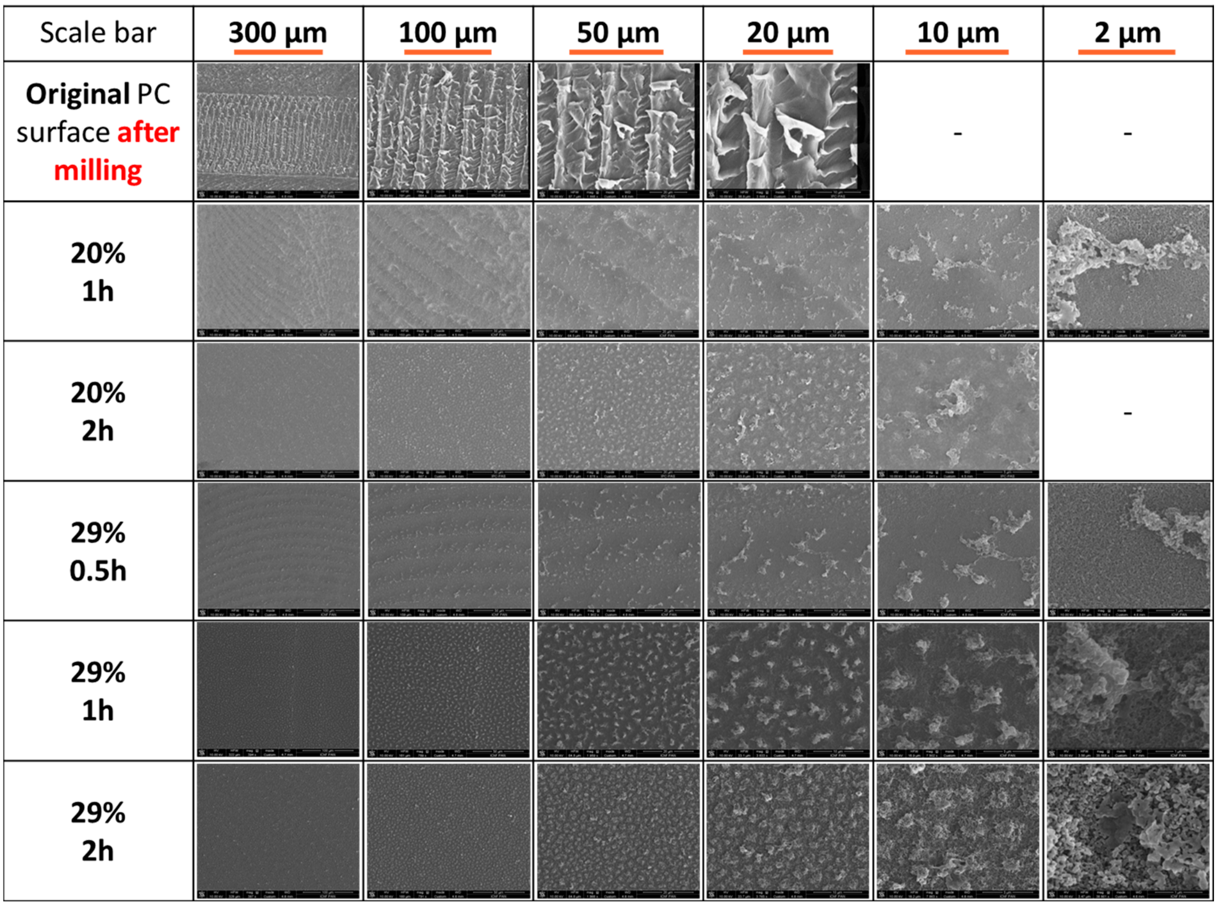

Figure S2 *Scanning electron microscopies of* ***milled*** *PC slabs that were modified with the use of various solutions of NH_4OH for various times at room temperature.*

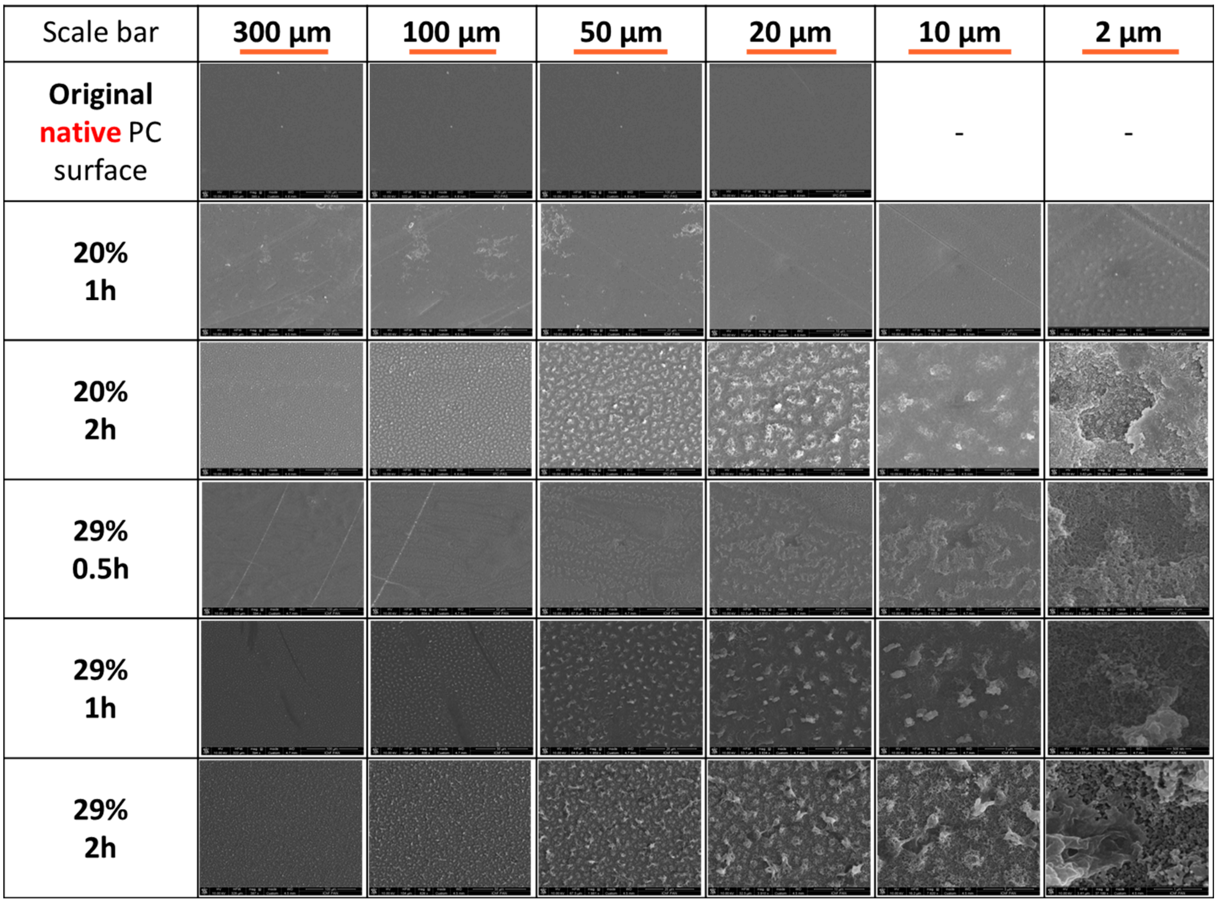

Figure S3. *Scanning electron microscopies of* ***native*** *PC slabs that were modified with the use of various solutions of NH_4OH for various times at room temperature.*
